# Cardioprotective effect of rosmarinic acid against myocardial ischaemia/reperfusion injury via suppression of the NF-κB inflammatory signalling pathway and ROS production in mice

**DOI:** 10.1080/13880209.2021.1878236

**Published:** 2021-02-18

**Authors:** Wei Quan, Hui-xian Liu, Wei Zhang, Wei-juan Lou, Yang-ze Gong, Chong Yuan, Qing Shao, Na Wang, Chao Guo, Fei Liu

**Affiliations:** a Xi’an Mental Health Center, School of Medicine, Xi’an Jiaotong University, Xi’an, China; b College of Pharmacy, Hebei University of Chinese Medicine, Shijiazhuang, China; c Department of Pharmacy, Xijing Hospital, Air Force Medical University, Xi’an, China; d Department of Pathology, School of Basic Medical Sciences, Fudan University, Shanghai, China

**Keywords:** Cardioprotection, inflammation response, oxoglutarate dehydrogenase, antioxidation

## Abstract

**Context:**

Rosmarinic acid (RosA), a natural poly-phenolic compound isolated from a variety of Labiatae herbs, has been reported to have a range of biological effects.

**Objective:**

To investigate the cardioprotective effects of RosA against myocardial ischaemia/reperfusion (I/R) injury.

**Materials and methods:**

Male C57BL/6J mice were given RosA (100 mg/kg) via intragastric administration. After 1 week of administration, the mice were subjected to 30 min/24 h myocardial I/R injury. The mice were randomly subdivided into 4 groups: Vehicle, RosA, Vehicle + I/R, and RosA + I/R. Infarct size (IS), cardiac function (including EF, FS), histopathology, serum enzyme activities, ROS changes, cis aconitase (ACO) activity, and specific mRNA and protein levels were assessed *in vivo*. HL-1 cells were pre-treated with or without RosA (50 μM), followed by stimulation with 9 h/6 h of oxygen and glucose deprivation/re-oxygenation (OGD/R). The cells were randomly subdivided into 4 groups: Vehicle, RosA, Vehicle + OGD/R, and RosA + OGD/R. Lactate dehydrogenase (LDH) levels, ACO activity, ROS changes and protein levels were measured *in vitro*.

**Results:**

Treatment with RosA reduced the following indicators *in vivo* (*p* < 0.05): (1) IS (14.5%); (2) EF (-23.4%) and FS (-18.4%); (3) the myocardial injury enzymes CK-MB (20.8 ng/mL) and cTnI (7.7 ng/mL); (4) DHE-ROS: (94.1%); (5) ACO activity (-2.1 mU/mg protein); (6) *ogdh* mRNA level (122.9%); and (7) OGDH protein level (69.9%). Moreover, treatment with RosA attenuated the following indicators *in vitro* (*p* < 0.05): (1) LDH level (191 U/L); (2) DHE-ROS: (165.2%); (3) ACO activity (-3.2 mU/mg protein); (4) *ogdh* mRNA level (70.0%); and (5) OGDH (110.1%), p-IκB-a (56.8%), and p-NF-κB (57.7%) protein levels.

**Conclusions:**

RosA has the potential to treat myocardial I/R injury with potential application in the clinic.

## Introduction

Acute myocardial infarction (AMI) is myocardial necrosis caused by acute, persistent ischaemia and hypoxia within the coronary arteries (Lai et al. 2020). Reperfusion treatment is the primary therapeutic intervention in the treatment of AMI. However, blood pressure drops, cardiac dysfunction and other phenomena that occur after blood flow recovery restore cardiac function but aggravate cardiac dysfunction and can induce structural impairment, such as myocardial ischaemia/reperfusion (I/R) injury (Frank et al. 2012). Studies have demonstrated that myocardial injury caused by reperfusion injury accounts for more than 50% of total myocardial injury following recovery of blood flow (Hausenloy and Yellon 2013). Exploration and discovery of novel drug targets, as well as new strategies for effective intervention, are popular and problematic issues that require urgent solutions.

**Figure 1.**
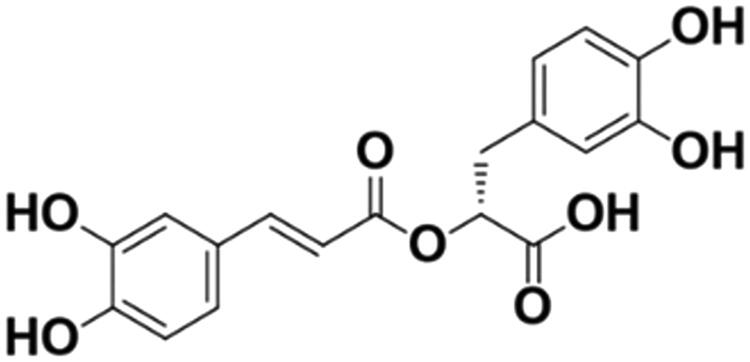
Chemical structure of rosmarinic acid (RosA). Molecular formula: C_18_H_16_O_8_; molecular weight: 360.32.

Rosmarinic acid (α-*O*-caffeoyl-3,4-dihydroxyphenyl lactic acid, RosA) (Figure 1) is a phenolic compound isolated from a variety of Labiatae herbs and has diverse immunoregulatory functions, such as antioxidant (Luft et al. 2019), anti-inflammatory (Rodriguez-Luna et al. 2019), antimicrobial (Benedec et al. 2015), antiviral (Swarup et al. 2007), antiallergic (Osakabe et al. 2004), antidiabetic (Berhow et al. 2012), antidepressant (Ito et al. 2008), and antitumor activities (Anusuya and Manoharan 2011). Although such functions have been reported, very little is known regarding the multitargeted action of RosA in treating cardiovascular disease, especially in myocardial I/R injury. Therefore, in the current study, RosA was tested *in vivo* in a myocardial I/R injury mouse model. Furthermore, *in vitro* studies of the HL-1 cardiomyocyte cell line subjected to simulated I/R were performed to investigate the therapeutic effects and underlying mechanisms of RosA.

## Materials and methods

### Materials and reagents

RosA was supplied by Shanghai Topscience Technology Co., Ltd. (Shanghai, China), and its purity (99.47%) was determined by high-performance liquid chromatography (HPLC). RosA is soluble in water and has a molecular formula of C_18_H_16_O_8_ and a molecular weight of 360.32.

MTT was purchased from China Pharmaceutical Group Shanghai Medical Devices Co., Ltd. (Shanghai, China). Evans blue was purchased from Solarbio Life Sciences Co., Ltd. (Beijing, China). The kits for determination of creatine kinase-MB (CK-MB), cardiac troponin I (cTnI) and lactate dehydrogenase (LDH) were obtained from Jiancheng Bioengineering Institute (Nanjing, China). Reactive Oxygen Species (ROS) Fluorescent Probe-Dihydroethidium (DHE) was obtained from Shanghai BestBio Biotechnology Co., Ltd. (Shanghai, China). An aconitase (ACO) assay kit was obtained from Abcam (MA, USA). DMEM (High Glucose/Low Glucose) and foetal bovine serum (FBS) were obtained from HyClone Laboratories (Thermo Fisher, Shanghai, China). Anti-p-NF-κB (p65), anti-NF-κB (p65), anti-p-IκB, and anti-IκB antibodies were obtained from Cell Signalling Technology (MA, USA). Anti-OGDH antibody was obtained from Abcam (MA, USA). Anti-β-actin antibody was obtained from Sigma (Deisenhofen, Germany). The RNA simple total RNA kit, FastKing RT kit and qPCR mix (SYBR Green I) were obtained from Tsingke Biological Technology (Beijing, China). Primers for *ogdh* and *gapdh* were designed by Tsingke Biological Technology (Beijing, China).

### Animals and myocardial I/R injury *in vivo*

C57BL/6J male mice that were 6–8 weeks old and weighed 18–22 g were purchased from the Animal Experimental Centre of the Air Force Medical University. All of the experiments were approved by the Ethics Committee of AFMU. The protocol was performed according to the Guide for the Care and Use of Laboratory Animals. Mice were anaesthetised with 2% isoflurane inhalation, a skin incision was made at the left chest, and a small hole was made at the fourth intercostal space. Subsequently, the pleural membrane and pericardium were opened with a clamp, and the heart was then gently ‘popped out’ through the hole. The left main descending coronary artery (LCA) was sutured and ligated using a 6–0 silk suture, and the heart was immediately replaced into the intrathoracic space following ligation. The mice were subjected to myocardial ischaemia for 30 min, followed by 24 h of reperfusion. Mice in the sham group underwent the same surgery, but the LCA was not occluded.

### Cell culture and simulated I/R injury *in vitro*

Cells of the cardiac muscle cell line HL-1 were cultured in DMEM supplemented with 10% FBS, streptomycin (100 μg/mL), and penicillin (100 U/mL) at 37 °C and 5% CO_2_. HL-1 cells were processed under conditions of oxygen glucose deprivation followed by reperfusion (OGD/R) to induce a simulated ischaemia/reperfusion injury model *in vitro*. Cells were exposed to hypoxia for 6 h in low-glucose DMEM, after which hypoxia was induced in a hypoxia incubator chamber saturated with a 5% CO_2_ and N_2_ balance. Non-OGD control groups were maintained at normoxia in high-glucose DMEM with 10% FBS. Subsequently, all of the sample groups were reoxygenated in a 21% O_2_/5% CO_2_/N_2_ balance and resupplied with nutrients in high-glucose DMEM with 10% FBS.

### Experimental groups

According to preliminary experimental data, 100 mg/kg of RosA was used *in vivo*, in accordance with previous studies in which 100 mg/kg of RosA was determined to confer a cardioprotective effect (Noguchi-Shinohara et al. 2015; Zhang et al. 2018). The mice were randomly subdivided into 4 groups: (1) Vehicle; (2) RosA; (3) Vehicle + I/R; and (4) RosA + I/R. Accordingly, 50 μM of RosA was used *in vitro*, in accordance with previous studies (Diao et al. 2016; Han et al. 2017; Zhang et al. 2019). The cells were then randomly subdivided into 4 groups: (1) Vehicle; (2) RosA; (3) Vehicle + OGD/R; and (4) RosA + OGD/R.

### Measurement of area at risk and infarct size

To obtain samples for infarct size analysis, mouse hearts were briefly perfused with 3% Evans blue in normal saline and then removed. Subsequently, the LV tissue was frozen and cut into 6 slices along the horizontal axis. The heart sections were then incubated with 1% TTC in phosphate buffer (pH 7.4, 37 °C) for half an hour in the dark. After staining with TTC, red areas in the heart indicated ischaemic but viable tissue, while pale areas represented infarcted myocardium. Images pertaining to Evans blue and TTC dyeing results were obtained with a macrolens. Infarct area sizes were determined using Image-Pro software. The size of the infarction area was calculated as the infarct area divided by the area at risk (IF/AAR).

### Echocardiographic measurements

Echocardiographic views were acquired with the Vevo2100 (Visual Sonics Int., Toronto, Canada) 24 h after surgery. The mice were anaesthetised with 2% isoflurane inhalation with an isoflurane delivery system (Viking Medical, Medford, NJ, USA) during echocardiographic examination. In M-mode, left ventricular end-diastolic diameter (LVEDD), left ventricular end-systolic diameters (LVESD), posterior wall diastolic thickness (PWT, d), and posterior wall systolic thickness (PWT, s) were measured (Gardin et al. 2002). GraphPad Prism software, version 5, was used to calculate the LV fractional shortening (FS) and ejection fraction (EF).

### Histopathological examination

AAR tissue sections were fixed in 4% paraformaldehyde and embedded into paraffin. Subsequently, they were cut into 5 μm thick sections for histopathological analysis. Paraffin-embedded sections were then stained with H&E, and morphological evaluation was performed via light microscopy. The extent of myocardial tissue injury was assessed in five random fields (×400 magnification).

### Measurement of CK-MB and cTnI release in serum

At the end of the experiment, blood was collected, and serum was separated by centrifugation. CK-MB and cTnI levels were measured spectrophotometrically using standard enzyme-linked immunosorbent assay kits and a microplate reader (Thermo Scientific, USA) according to the manufacturer’s instructions.

### Measurement of LDH release in culture medium

LDH activity was measured spectrophotometrically using standard enzyme-linked immunosorbent assay kits and a microplate reader (Thermo Scientific, USA) according to the manufacturer’s instructions.

### Measurement of ROS production

ROS production was detected using an ROS-sensitive fluorescent indicator DHE assay kit. ROS generation was represented by the total DHE fluorescence, and fluorescence intensity was detected with a fluorescence microscope.

### Measurement of ACO activity

Enzyme activity was evaluated in 800 *g* of supernatant from heart extract (initial recommendation = 40 mg) or cell extract (initial recommendation = 1 × 10^6^ cells). The reaction mix was added and incubated at 25 °C for 60 min, after which the developer was added and incubated at 25 °C for 10 min. ACO activity was determined using a microplate reader at an optical density of 450 nm. The absorbance (A1) at 240 nm was then measured for 10 s, and the sample was quickly placed at 25 °C for 5 min, after which the cuvette was quickly removed and wiped dry. The absorbance (A2) was recorded at 5 min, and ΔA = A1-A2 was calculated. Additionally, the activity of ACO was computed according to the sample protein concentration (mU/mg prot) = 555.55 × ΔA ÷ Cpr × N. Cpr1: sample protein concentration, mg/mL; N: dilution factor; ΔA: difference in absorbance between two times.

### RT-PCR

Total RNA was extracted using a Total RNA Kit (Tsingke Biotechnology, Beijing, China). Reverse transcription (RT) of mRNA was conducted via a standard reaction using a FastKing RT Kit (Tsingke Biotechnology, Beijing, China). Real-time PCR was performed using Fast qPCR Mix (SYBR Green I) (Tsingke Biological Technology, Beijing, China). The RT-PCR reaction was performed in a 20 μL system containing cDNA template and 10 μM forward and reverse primers. Primers for *ogdh* and *gapdh* were designed (Table 1). RT-qPCR was performed using an ABI StepOnePlus system (Applied Biosystems) followed by a melting curve analysis with the following cycling program: initial activation at 95 °C for 2 min, followed by 48 cycles of denaturation at 95 °C for 15 s, annealing at 55 °C for 30 s, and extension at 72 °C for 15 s. The results for each gene were normalized to Gapdh messenger RNA (mRNA) levels, which were measured in parallel in each sample.

**Table 1.** Primers for *Ogdh* and *Gapdh* showed in the table.

Gene	Primer sequence
*Gapdh*	F:5′CACTGAGCAAGAGAGGCCCTAT 3′ R:5′GCAGCGAACTTTATTGATGGTATT 3′
*Ogdh*	F:5′CTCAGCCTTGTGGAGGTGGA 3′ R:5′CTCATCGCTAGATGTATGGTTCA 3′

### Western blotting assay

The protein concentration of each sample was measured using a Bio-Rad Protein Assay Kit (Bio-Rad Laboratories Inc., Hercules, CA, USA). Proteins were separated via 10% sodium dodecyl sulphate-polyacrylamide gel electrophoresis (SDS-PAGE) and transferred onto polyvinylidene difluoride (PVDF) membranes. The membranes were blocked with 5% non-fat dried milk at room temperature for 2 h and then with primary antibodies (targeting p-NF-κB, p-IκB-a, NF-κB, IκB-a, OGDH and β-actin) at 4 °C overnight. The membranes were then incubated with HRP-conjugated secondary antibody for 1 h at room temperature. Immunolabeled proteins were detected using ECL-Plus reagent, and blots were analyzed using Quantity One software (Bio-Rad Laboratories Inc., Hercules, CA, USA).

### Molecular docking

ChemBioDraw Ultra software, version 17.0, was used to draw the structure of RosA, which was then converted into a three-dimensional structure and optimized using the MMFF94 force field. The three-dimensional structure of RelA (transcription factor p65, also known as nuclear factor NF-κB p65 subunit, a protein encoded by the *RelA* gene in humans) was downloaded from the RCSB Protein Data Bank. The three-dimensional structure of RelA (PDB ID: 6GGR) was used as the protein for docking in this project. Both NF-κB and RosA were converted into PDBQT format using AutodockTools software, version 1.5.6. Autodock Vina software, version 1.1.2, was used for molecular docking research. The coordinates of the NF-κB active site were as follows: centre_X= −10.733, centre_Y = 12.416, centre_Z = 68.829; size_X = 20, size_Y = 20, and size_Z = 20. To increase accuracy, the parameter exhaustiveness was set to 20. Unless otherwise specified, all of the other parameters were set to default values. Finally, conformation with the highest score was selected, and the results were analyzed using Free Maestro software, version 11.9.

### Statistical analysis

Data are presented as the mean ± S.D. and were analyzed using GraphPad Prism software (La Jolla, CA, USA). One-way ANOVA followed by Tukey’s test was applied for statistical analysis of the parameters. A value of *p* < 0.05 was considered statistically significant.

## Results

### Effects of RosA on myocardial infarct size

**Figure 2.**
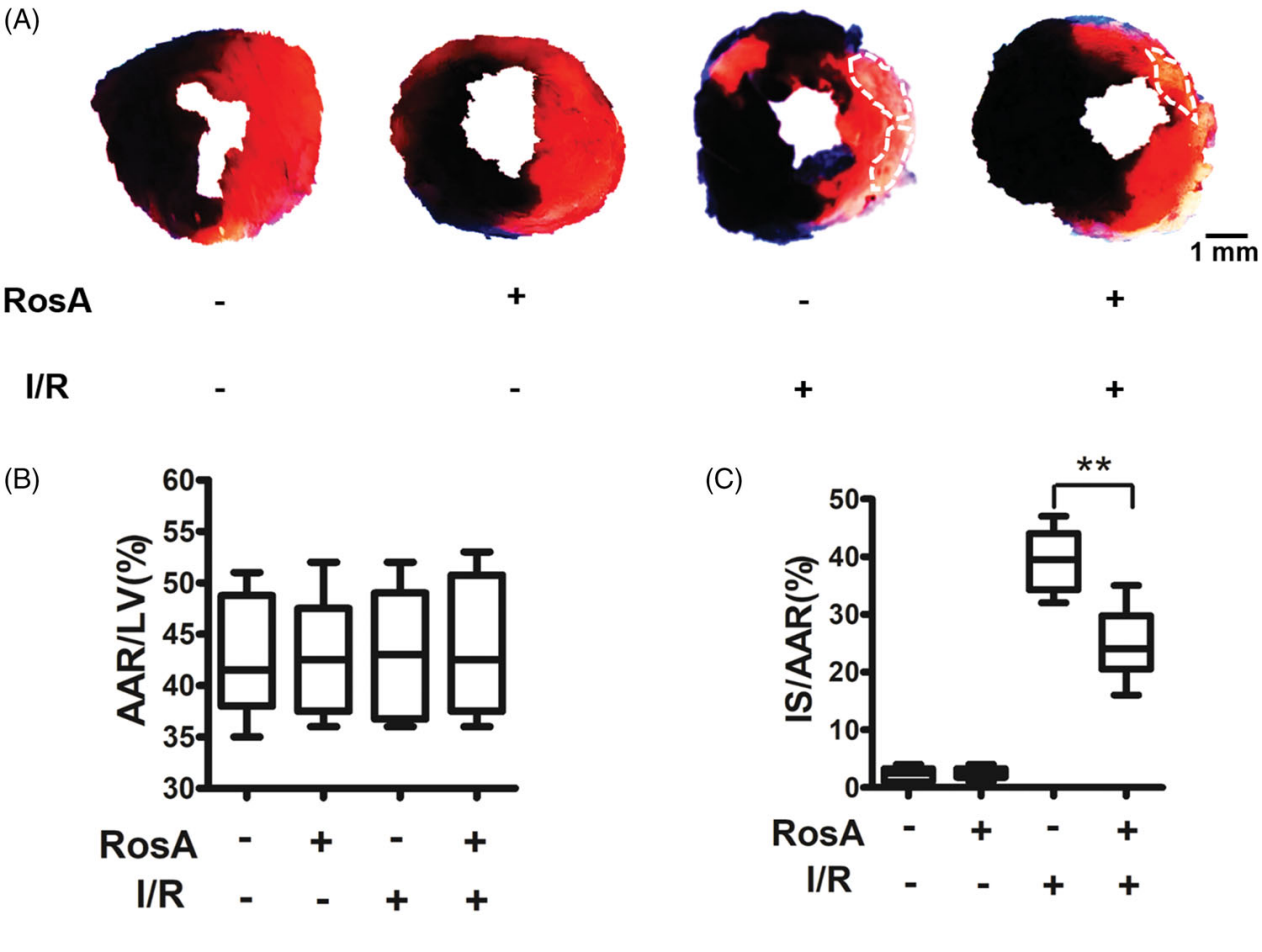
Effects of RosA on myocardial infarct size. (A) Representative photomicrographs of heart sections stained with Evans blue and TTC for different treatment groups are shown. (B) AAR/LV was similar among groups. (C) Treatment with RosA markedly reduced the infarct size caused by myocardial I/R injury. Data are expressed as the mean ± S.D. (*n* = 6). Significance was determined by ANOVA followed by Tukey’s test. **p < 0.01 vs. Vehicle + I/R. AAR: area at risk; LV: left ventricle; IS: infarct size.

No significant difference was observed in AAR/LV (%) between the vehicle- and RosA-treated groups, indicating that a comparable degree of ischaemic jeopardy was present between the groups following occlusion of the left anterior descending artery (LAD; Figure 2(B)). However, treatment with 100 mg/kg RosA significantly reduced the IS/AAR (%) compared to the I/R + Vehicle group (24.83 ± 6.46% vs. 39.33 ± 5.61%, Figure 2(C)).

### Effects of RosA on cardiac dysfunction

**Figure 3.**
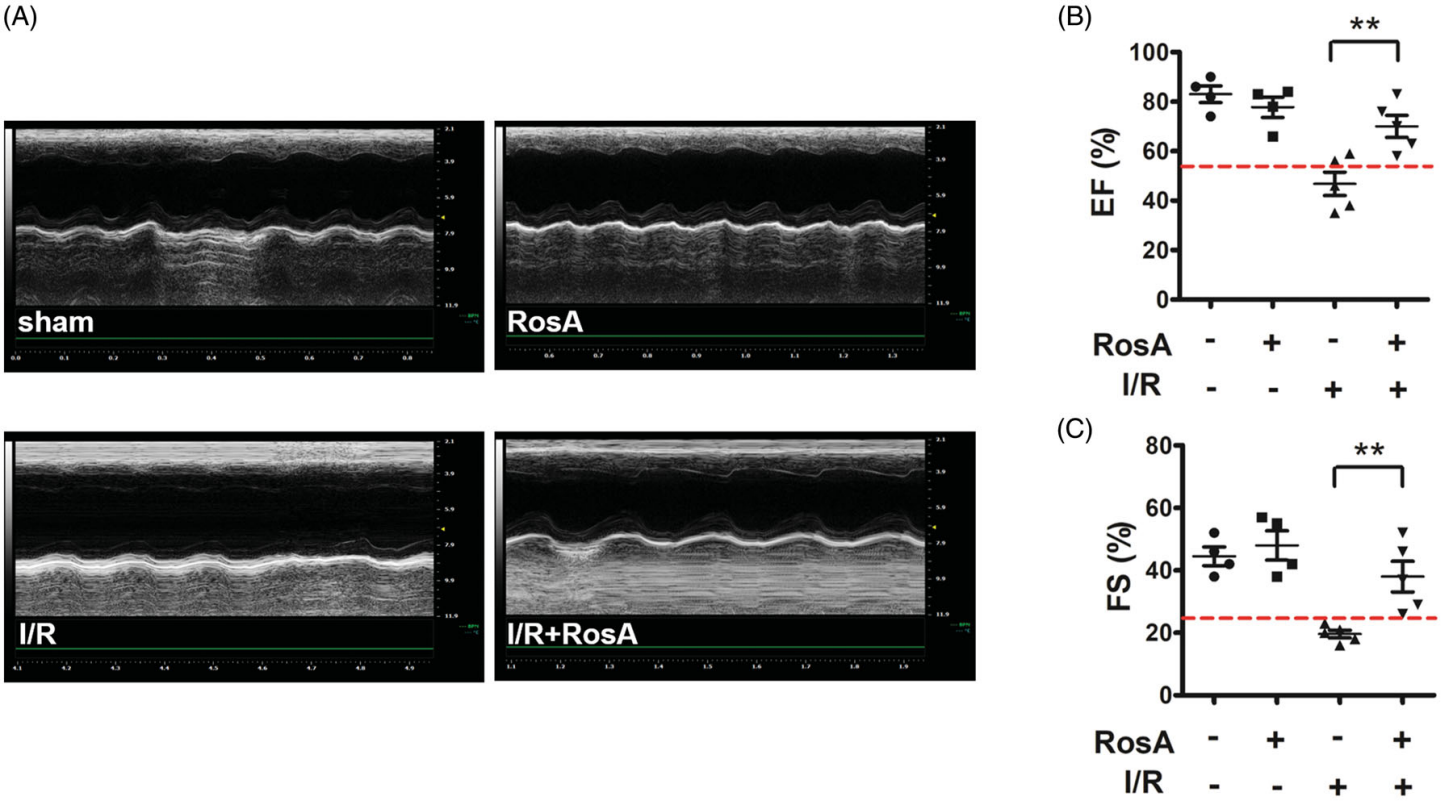
Effects of RosA on cardiac dysfunction. (A) Representative echocardiographs showing cardiac function from the various groups. (B) EF and (C) FS measured by echocardiography. Data are expressed as the mean ± S.D. (*n* = 4–5). Significance was determined by ANOVA followed by Tukey’s test. **p < 0.01 vs. Vehicle + I/R. EF: ejection fraction; FS: fractional shortening.

After I/R treatment, echocardiographic examinations were used as non-invasive methods to detect mouse cardiac function. Normal cardiac function can be evaluated by EF (EF ≥ 55%) (Sweitzer et al. 2008) and FS (FS ≥ 25%) (Gardin et al. 2002). As shown in Figure 3(A), representative images of the M-mode echocardiography indicated that the Vehicle + I/R group showed impaired cardiac function compared to the sham group. RosA markedly increased the levels of EF (70.2 ± 10.0% vs. 46.8 ± 10.6%, Figure 3(B)) and FS (38.0 ± 11.0% vs. 19.6 ± 2.7%, Figure 3(C)).

### Effects of RosA on cell necrosis

**Figure 4.**
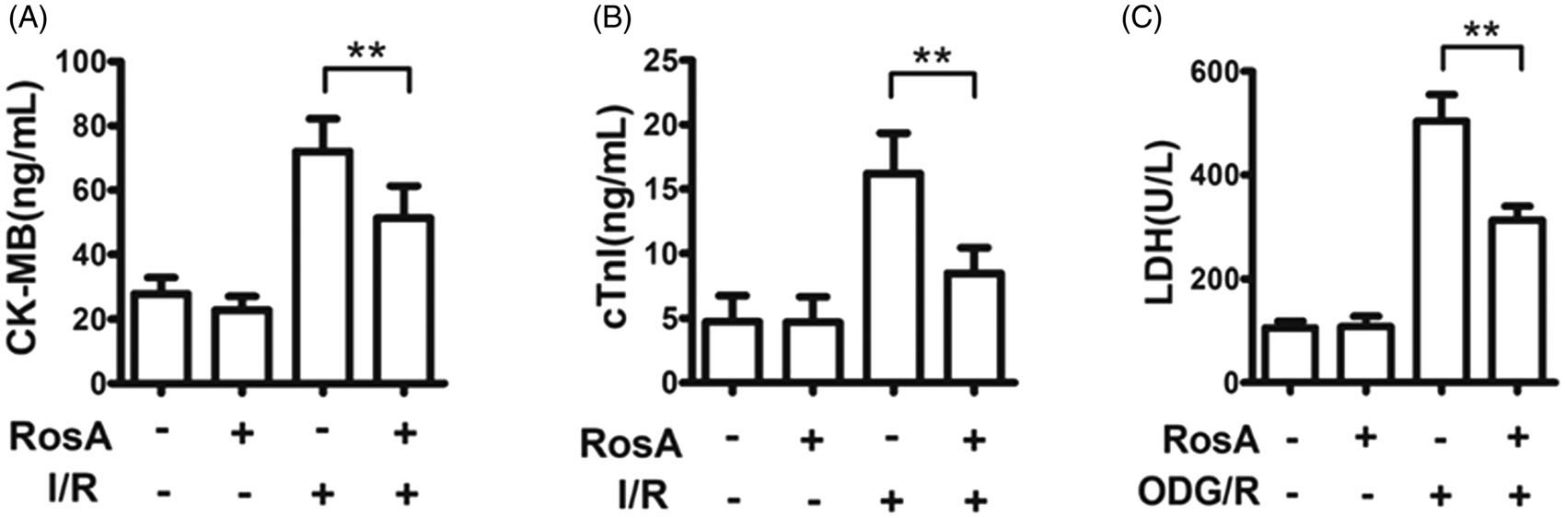
Effect of RosA on levels of CK-MB, cTnI and LDH for each group. (A) RosA reduces CK-MB levels in serum. (B) RosA reduces cTnI levels in serum. (C) RosA reduces LDH levels in culture medium. Data are expressed as the mean ± S.D. (*n* = 6). Significance was determined by ANOVA followed by Tukey’s test. **p < 0.01 vs. Vehicle + I/R or Vehicle + OGD/R.

A significant decrease in CK-MB and cTnI activities was observed in the RosA + I/R groups compared to the Vehicle + I/R group (CK-MB: 51.3 ± 10.0 ng/mL vs. 72.1 ± 10.2 ng/mL, Figure 4(A); cTnI: 5 ± 2.0 ng/mL vs. 16.2 ± 3.1 ng/mL, Figure 4(B)). At the same time, a significant decrease in LDH levels was observed in the RosA + OGD/R group compared to the Vehicle + OGD/R group (312.9 ± 26.9 U/L vs. 503.8 ± 51.4 U/L, Figure 4(C)).

### Effects of RosA on cardiac histopathology

**Figure 5.**
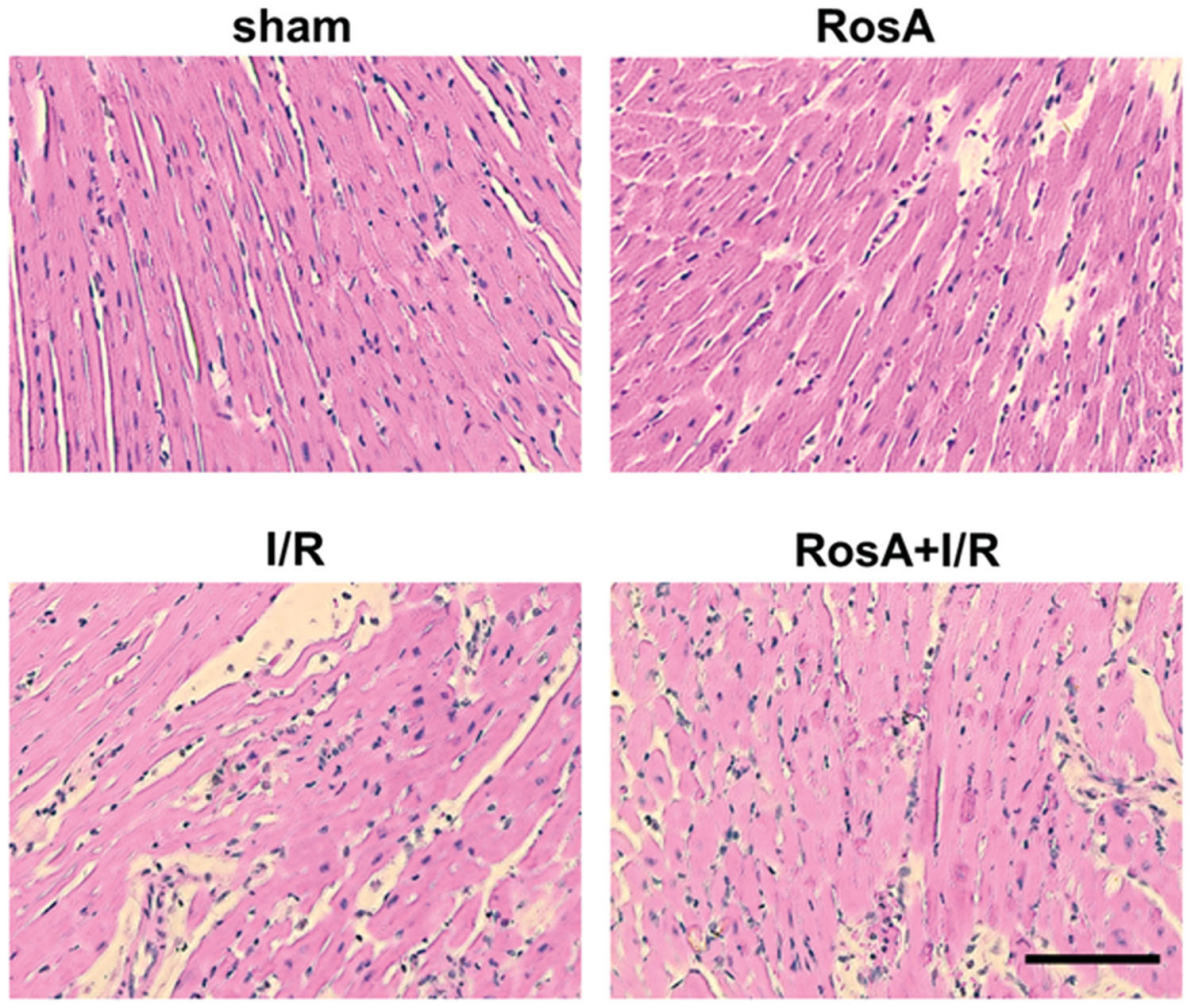
Representative light microscopic images of myocardial histopathological morphology (H&E, ×400).

The Vehicle + I/R group exhibited widespread myocardial structural damage, diffuse cloudy swelling, red-blood-cell extravasation, and infiltration of inflammatory cells. Subsequently, these myocardial injuries were ameliorated in the RosA treatment groups (Figure 5).

### Effects of RosA on ROS production

**Figure 6.**
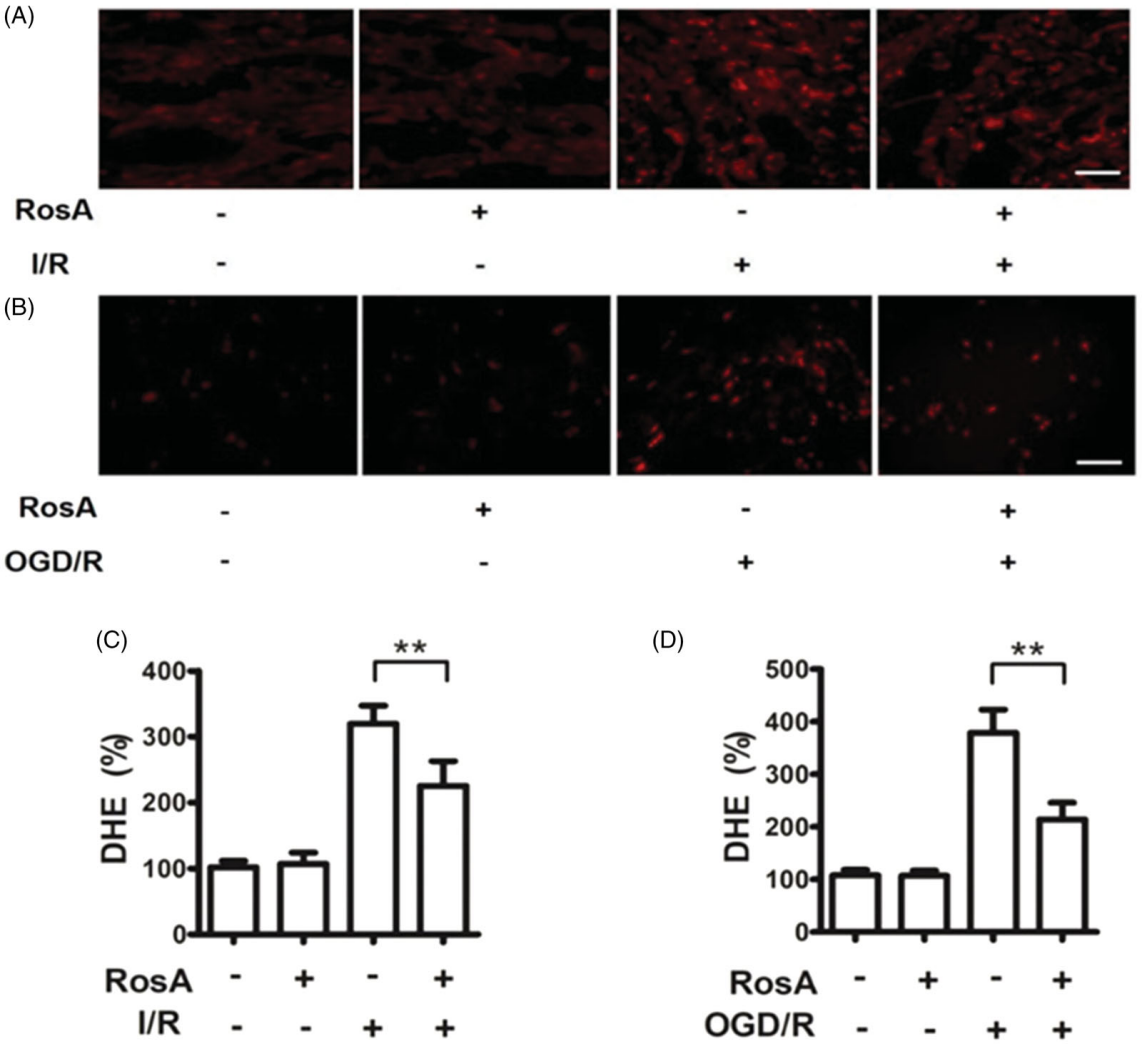
Effects of RosA on ROS production. (A) Representative micrograph of RosA reducing ROS production in myocardial I/R area. Scale bar, 100 μm. (B) Representative micrographs of RosA reducing ROS production after OGD/R injury in cells. (C) Statistical results of RosA reducing ROS production in myocardial I/R area. (D) Statistical results of RosA reducing ROS production after OGD/R injury in cells. Data are expressed as the mean ± S.D. (*n* = 3). Significance was determined by ANOVA followed by Tukey’s test. **p < 0.01 vs. Vehicle + I/R or Vehicle + OGD/R.

ROS changes could reflect the status of oxidative function, which can be a measure of oxidative tissue and cell damage (Li et al. 2019). The results of DHE-ROS measurement revealed that RosA could significantly reduce ROS production in myocardial I/R injury areas (225.6 ± 37.1% vs. 319.7 ± 27.1%, Figure 6(A,C)). Additionally, RosA was found to reduce ROS production following OGD/R injury in cells (213.9 ± 32.2% vs. 379.1 ± 44.2%, Figure 6(B,D)).

### Effects of RosA on oxidative inactivation of ACO

**Figure 7.**
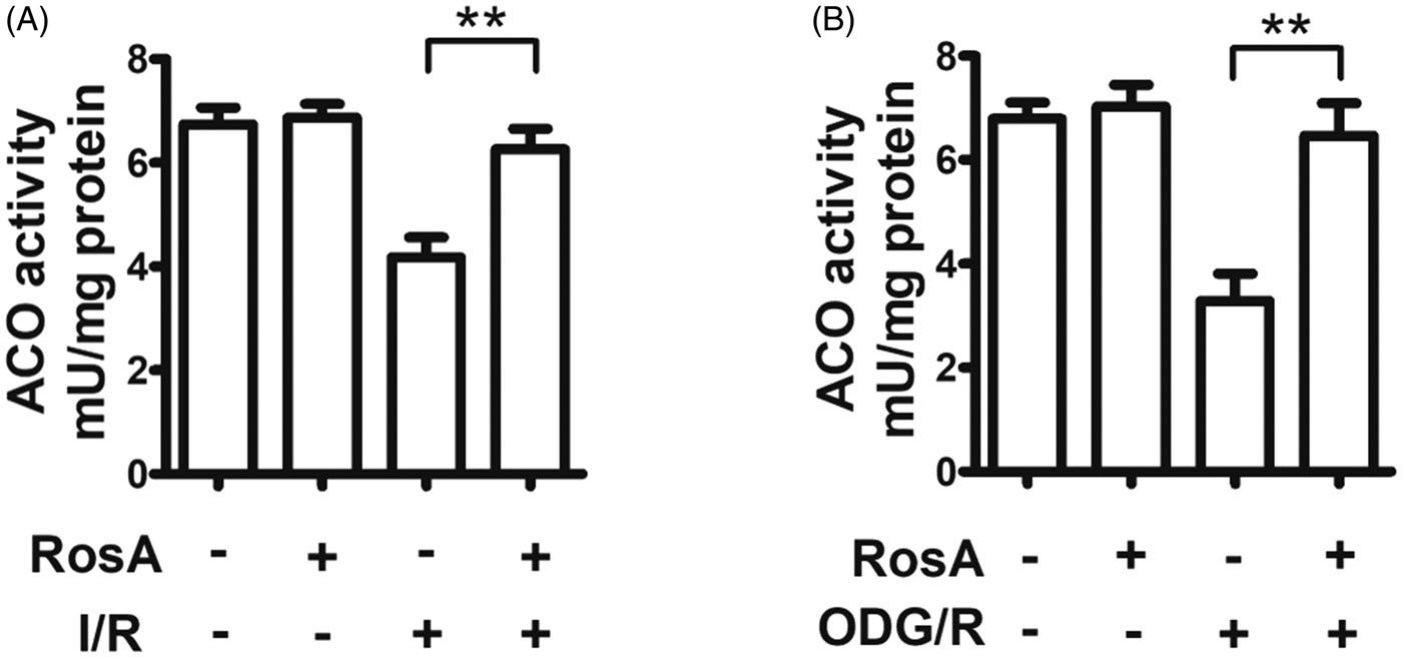
Effects of RosA on oxidative inactivation of ACO. (A) Statistical results of RosA reducing the oxidative inactivation of ACO activity in myocardial I/R area. (B) Statistical results of RosA reducing the oxidative inactivation of ACO activity after OGD/R injury in cells. Data are expressed as the mean ± S.D. (*n* = 3). Significance was determined by ANOVA followed by Tukey’s test. **p < 0.01 vs. Vehicle + I/R or Vehicle + OGD/R.

ACO is an important Fe-S protease found in cells. When its Fe-S active centre is damaged by myocardial I/R injury, it is attacked by ROS, which inactivate ACO (Sami et al. 2019). Here, RosA was found to reduce oxidative inactivation of ACO in the myocardial I/R injury area in mice (7.6 ± 0.4 mU/mg protein vs. 4.2 ± 0.4 mU/mg protein, Figure 7(A)). RosA was also observed to reduce oxidative inactivation of ACO following OGD/R injury in cells (6.3 ± 0.6 mU/mg protein vs. 3.1 ± 0.5 mU/mg protein, Figure 7(B)).

### Effects of RosA on ogdh mRNA and protein levels

**Figure 8.**
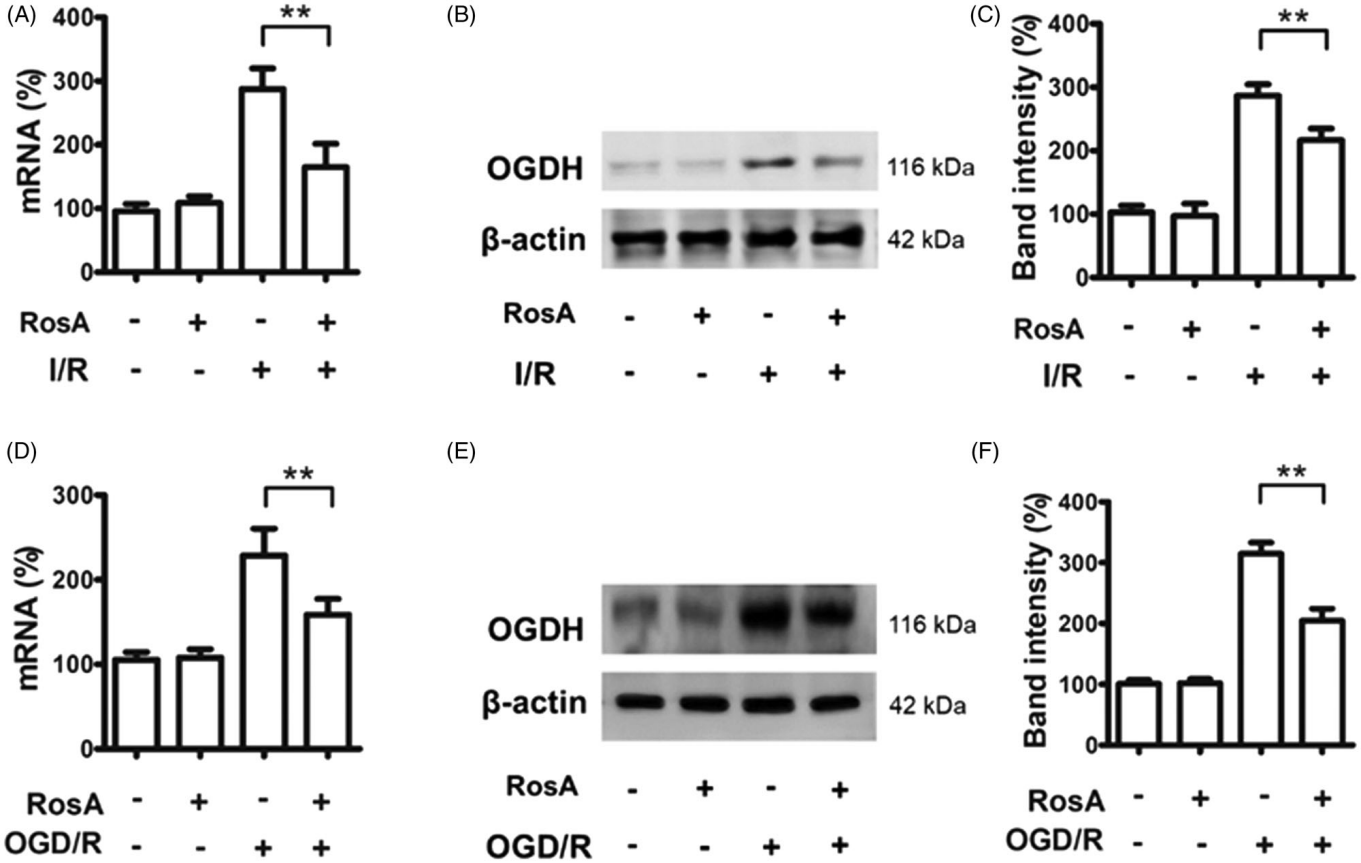
RosA reduced ogdh mRNA and protein levels. (A) RosA reduces ogdh mRNA levels in myocardial I/R area in mice. (B) RosA reduces OGDH protein levels in myocardial I/R area in mice. (C) Statistical results of RosA reducing OGDH protein levels in myocardial I/R area in mice. (D) RosA reduces ogdh mRNA levels after OGD/R injury in cells. (E) RosA reduces OGDH protein levels after OGD/R injury in cells. (F) Statistical results of RosA reducing OGDH protein levels after OGD/R injury in cells. Data are expressed as the mean ± S.D. (*n* = 3). Significance was determined by ANOVA followed by Tukey’s test. **p < 0.01 vs. Vehicle + I/R or Vehicle + OGD/R.

OGDH is vital for Krebs cycle metabolism and is a source of ROS (Mailloux et al. 2016a). RosA reduced *ogdh* mRNA (164.8 ± 36.8% vs. 287.7 ± 32.0%, Figure 8(A)) and protein (216.5 ± 18.4% vs. 286.4 ± 18.0%, Figure 8(B,C)) levels in the area of myocardial I/R injury. RosA also reduced *ogdh* mRNA (158.7 ± 18.6% vs. 228.7 ± 31.7%, Figure 8(D) and protein (204.7 ± 19.9% vs. 314.8 ± 18.5%, Figure 8(E,F)) levels after OGD/R injury in cells.

### Effects of RosA on the NF-κB signalling pathway

**Figure 9.**
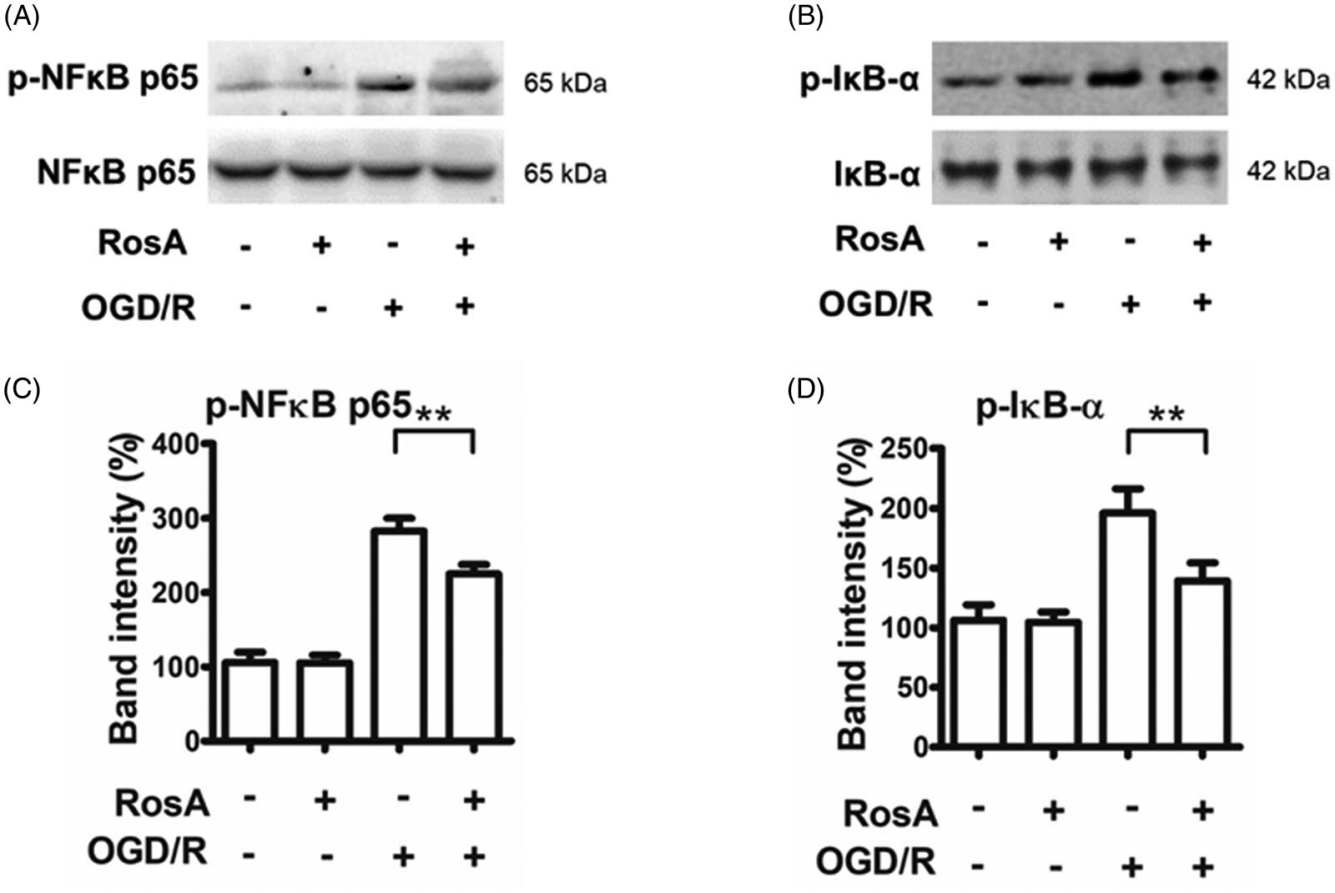
RosA reduced the protein levels of p-NFκB and p-IκB-α. (A) RosA reduced the protein level of p-NFκB. (B) Statistical results of RosA reducing the protein level of p-NFκB. (C) RosA reduced the protein levels of p-IκB-α. (D) Statistical results of RosA reducing the protein level of p-IκB-α. Data are expressed as the mean ± S.D. (*n* = 3). Significance was determined by ANOVA followed by Tukey’s test. **p < 0.01 vs. Vehicle + OGD/R.

Previous studies have shown that genes regulated by NF-κB could play a critical role in regulating ROS levels in cells (Liu et al. 2020). RosA reduced the protein levels of p-NFκB (225.2 ± 12.7% vs. 282.9 ± 17.1%, Figure 9(A,B)) and p-IκB-α (139.3 ± 15.1% vs. 196.1 ± 20.6%, Figure 9(C,D)) following OGD/R injury in cells.

### Key interactions between RosA and NF-κB protein

**Figure 10.**
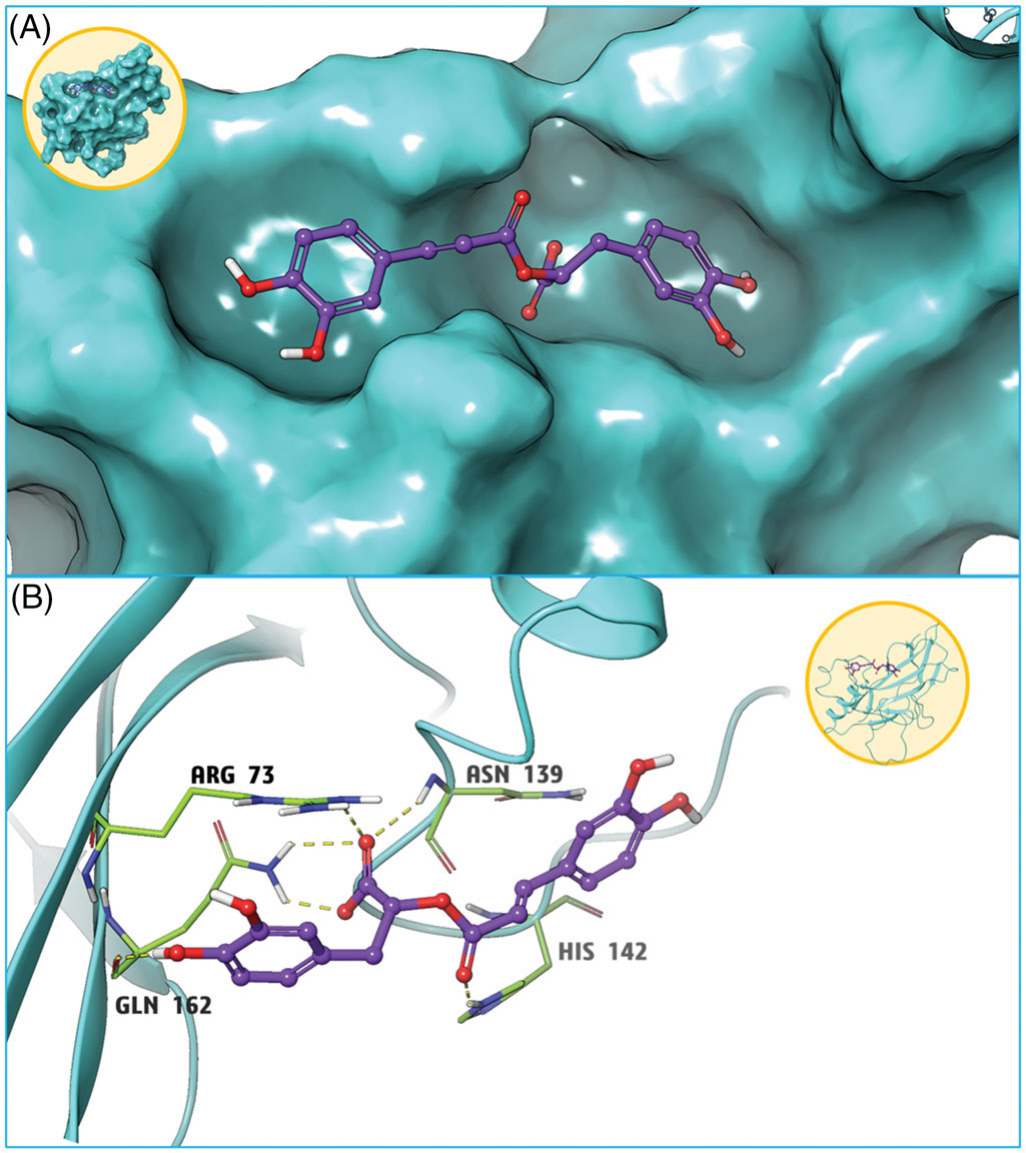
Molecular docking results of compound RosA and NFκB protein. (A) Small molecule RosA binds tightly with NFκB protein. (B) Hydrogen bond interaction is the main force between compound RosA and NFκB protein.

Utilizing the AutoDock Vina software, the free energy of the RosA compound bound to the active cavity of NF-κB p65 (RelA) protein was determined to be −7.2 kcal/mol. It is generally believed that, when the absolute value is greater than 7, the compound and protein are more likely to bind (Nagasundaram et al. 2016). The combination mode is shown in Figure 10(A). Accordingly, RosA was found to bind to the active site of the NF-κB p65 protein, forming six hydrogen bonds with the four amino acids ARG73, GLU162, ASN139, and HIS142 near the active site. These hydrogen bonding interactions served as the most important forces between the NF-κB p65 protein and RosA (Figure 10(B)).

## Discussion

RosA is a natural, water-soluble phenolic acid compound that can be isolated from *Rosmarinus officinalis* L. (Labiatae). Due to its widespread distribution, it is particularly found in plants of Lamiaceae and Boraginaceae in large amounts. RosA also serves as an effective active ingredient of *Salvia miltiorrhiza* Bge., *Perilla frutescens* (L.) Britt., *Prunella vulgaris* L. and other Chinese herbal medicines (Cai et al. 2019). Moreover, it possesses oxidation resistance (Zych et al. 2019), anti-inflammation (Fan et al. 2015), immune regulation (Cao et al. 2019) and anti-thrombosis (Zou et al. 1993) activities and other biological functions. Studies have found that RosA can help prevent cell damage caused by free radicals due to its strong antioxidant activity, which is related to its structure (Fujimoto et al. 2010). The catechol hydroxyl eliminates free radical activity, while its C-3 conjugated double bond has a synergistic effect (Imai et al. 2019). According to numerous investigations, oxidative stress is the most serious and long-lasting factor that leads to myocardial I/R injury (Gonzalez-Montero et al. 2018). Therefore, it is believed that RosA is a likely drug candidate for preventing and treating heart and cerebrovascular diseases. Previous studies have also reported that RosA has protective effects against acute ischaemic injury (Ramalho et al. 2014) and hypoxia/reoxygenation myocardial cell injury in rats (Zhang et al. 2017). Furthermore, recent studies have shown that RosA has strong anti-apoptotic ability and can protect cells from hydrogen peroxide-induced DNA damage and apoptosis (Salimei et al. 2007). In light of the above studies, this investigation assumed that RosA possesses protective effects in myocardial ischaemia/reperfusion injury.

In this study, we verified at the whole animal level that RosA has protective effects against ischaemia/reperfusion (I/R) injury. Infarct size and cardiac function are clinically regarded as the main indices in evaluating the degree of myocardial I/R injury (Touboul et al. 2015). According to this study, RosA could reduce the myocardial infarction area, improve heart function, and relieve pathological injury in the myocardial infarction area. Additionally, myocardial cell arrangement in the RosA + I/R group was found to be relatively sparse, with clear stripes. No expansion of blood vessels or infiltration of inflammatory cells were found in the mesenchyme. Myocardial enzymes can serve as common indicators for the diagnosis of myocardial injury, and CK-MB is considered a diagnostic indicator of acute myocardial infarction (Yildiz et al. 2014). cTnI is a regulatory protein in myocardial contraction and has greater sensitivity and specificity to myocardial injury than other myocardial enzymes (Wang et al. 2017). In addition, LDH is a marker of myocardial injury (Jie et al. 2019). RosA was found to significantly reduce these specific myocardial enzyme levels. Overall, RosA was shown to have significant protective effects in myocardial I/R injury.

The body’s oxidative function can be reflected by changes in ROS. ROS determination can be used to measure oxidative tissue and cell damage (Qiu et al. 2019), and previous studies have reported that RosA has a powerful antioxidation effect. Thus, DHE-ROS was adopted to detect the oxidation state of myocardial tissue and cells. Simply stated, the DHE-ROS detection kit detects active oxygen utilizing the fluorescent probe dihydroethidium. DHE is freely accessible to the cell through the living cell membrane and is oxidized by ROS in the cell to form ethidium oxide, which can mix with chromosomal DNA to produce red fluorescence. It is possible to estimate the amount of ROS and change in its content in cells according to the red fluorescence produced in living cells (Hardy et al. 2015). Accordingly, the obtained results demonstrated that RosA could reduce ROS generation in the myocardial I/R injury area and after cell OGD/R injury. Aconitase (ACO) is an important iron-sulfur (Fe-S) protease in cells that is attacked by ROS when its Fe-S active centre undergoes I/R injury, leading to ACO oxidative inactivation (Lou et al. 2014). According to aconitase detection, RosA was found to alleviate oxidative inactivation of ACO in the myocardial I/R injury area in mice and after cell OGD/R injury. The above experiments verified that the myocardial protective effect of RosA is closely correlated with a reduction in ROS generation.

Ketoglutarate dehydrogenase (OGDH) is a key control point in the tricarboxylic acid cycle and serves as a main source of ROS generation in cells (Mailloux et al. 2016a, 2016b). qPCR and western blotting analysis showed that RosA reduced *ogdh* mRNA and protein levels following myocardial I/R injury in mice and reduced *ogdh* mRNA and protein levels following OGD/R injury in cardiomyocytes, suggesting that RosA could inhibit OGDH to reduce ROS generation in tissues and cells.

NF-κB p65 (RelA) is a primary member of the NF-κB family and is a nuclear transcription factor for the NF-κB signalling pathway (Liu et al. 2019). In the unstimulated state, p65, under regulation of the inhibitory factor IκB, forms a complex dimer located in the cytoplasm, existing in a resting state. After the cell is exposed to an external stimulus that leads to enzymatic dissociation of the inhibitor IκB, NF-κB p65 is activated and released. After nuclear transfer, it can be combined with the promoter region of the ogdh gene to initiate and regulate gene transcription (Shen et al. 2017). Therefore, the expression levels of p-NF-κB and p-IκB were detected at the cellular level, and it was found that RosA could reduce the expression levels of the key proteins p-NF-κB and p-IκB in the NF-κB signalling pathway following OGD/R injury.

To further confirm the interaction between RosA and NF-κB protein, AutoDock Vina software was used for molecular docking. Here, it was found that the free energy of the active cavity of RosA and the key protein NF-κB p65 was −7.2 kcal/mol, suggesting that RosA was closely bound to the surface of the key protein NF-κB p65, with adequate spatial matching between the two. Moreover, RosA bound to the active site of NF-κB p65, forming six hydrogen bond interactions with the four amino acids ARG73, GLU162, ASN139 and HIS142 near the active site. These hydrogen bond interactions constitute the main acting force between NF-κB protein and RosA, thus forming a stable compound. The aforementioned molecular docking study could provide a reasonable explanation for the interaction between NF-κB protein and RosA.

## Conclusions

In conclusion, this study boldly speculates that RosA might affect the activation and nuclear translocation of NF-κB by binding to the NF-κB protein, inhibiting the *ogdh* mRNA level and OGDH protein level and thereby reducing ROS generation in tissues and cells, ultimately alleviating myocardium I/R injury.
